# The Role of Calcitonin Gene Related Peptide (CGRP) in Neurogenic Vasodilation and Its Cardioprotective Effects

**DOI:** 10.3389/fphys.2018.01249

**Published:** 2018-09-19

**Authors:** Zizheng Kee, Xenia Kodji, Susan D. Brain

**Affiliations:** Section of Vascular Biology & Inflammation, BHF Centre for Cardiovascular Research, School of Cardiovascular Medicine and Sciences, King’s College London, London, United Kingdom

**Keywords:** CGRP, neurogenic vasodilation, rodents, humans, hypertension, heart failure

## Abstract

Calcitonin gene-related peptide (CGRP) is a highly potent vasoactive peptide released from sensory nerves, which is now proposed to have protective effects in several cardiovascular diseases. The major α-form is produced from alternate splicing and processing of the calcitonin gene. The CGRP receptor is a complex composed of calcitonin like receptor (CLR) and a single transmembrane protein, RAMP1. CGRP is a potent vasodilator and proposed to have protective effects in several cardiovascular diseases. CGRP has a proven role in migraine and selective antagonists and antibodies are now reaching the clinic for treatment of migraine. These clinical trials with antagonists and antibodies indicate that CGRP does not play an obvious role in the physiological control of human blood pressure. This review discusses the vasodilator and hypotensive effects of CGRP and the role of CGRP in mediating cardioprotective effects in various cardiovascular models and disorders. In models of hypertension, CGRP protects against the onset and progression of hypertensive states by potentially counteracting against the pro-hypertensive systems such as the renin-angiotensin-aldosterone system (RAAS) and the sympathetic system. With regards to its cardioprotective effects in conditions such as heart failure and ischaemia, CGRP-containing nerves innervate throughout cardiac tissue and the vasculature, where evidence shows this peptide alleviates various aspects of their pathophysiology, including cardiac hypertrophy, reperfusion injury, cardiac inflammation, and apoptosis. Hence, CGRP has been suggested as a cardioprotective, endogenous mediator released under stress to help preserve cardiovascular function. With the recent developments of various CGRP-targeted pharmacotherapies, in the form of CGRP antibodies/antagonists as well as a CGRP analog, this review provides a summary and a discussion of the most recent basic science and clinical findings, initiating a discussion on the future of CGRP as a novel target in various cardiovascular diseases.

## Introduction

Calcitonin gene-related peptide (CGRP) was first identified in 1982 (Amara et al., 1982). It was soon established that the neuropeptide is a potent vasodilator and a transmitter in the peripheral and central nervous systems (Rosenfeld et al., 1983; Brain et al., 1985), resulting in their predicted roles in pain and cardiovascular regulation (Rosenfeld et al., 1983). It was soon realized that CGRP release in trigeminal neurons is associated with control of cerebral vascular tone and plays a role in migraine (O’Connor and van der Kooy, 1988; Edvinsson et al., 2012). As such, drugs targeting CGRP are currently being investigated for use clinically for the treatment of migraine. The first non-peptide CGRP antagonist, BIBN 4096 BS, was shown to be effective in animals and humans (Doods et al., 2000; Olesen et al., 2004). There are currently a number of CGRP monoclonal antibodies and antagonists in development which are showing good efficacy for migraine, with up to 32% of patients being completely relieved of symptoms. These drugs are currently undergoing approval for the treatment of migraine. However, as evidence suggests that CGRP possesses protective properties in various cardiovascular diseases (Smillie et al., 2014; Aubdool et al., 2017), there are concerns regarding cardiovascular safety despite minimal cardiovascular issues being observed in clinical trials to date (Paemeleire and MaassenVanDenBrink, 2018; Raffaelli and Reuter, 2018). This review aims to summarize knowledge on the potential role of CGRP in cardiovascular disease to aid the discussion of potential adverse effects of anti-CGRP treatments as well as to guide the developments of novel CGRP-dependent targets for various cardiovascular conditions.

## Cgrp and Receptor

### CGRP Synthesis and Structure

**FIGURE 1:**
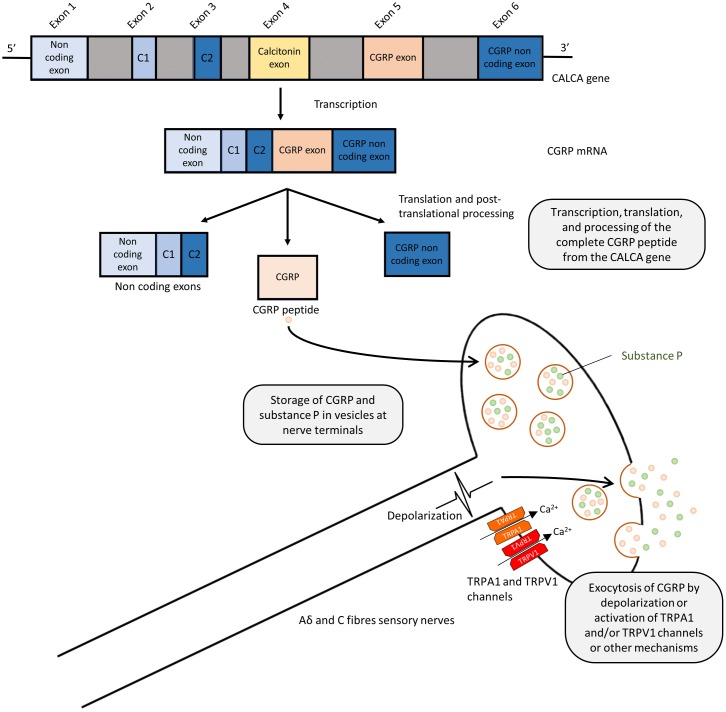
Splicing and post-translational processing of the complete CGRP peptide from the CALCA gene, vesicular packaging, and release from sensory nerves. Exons 1,2,3,5, and 6 of the CALCA gene are transcribed to form the CGRP mRNA which is then translated to protein. Post-translational cleavage produces the complete 37-amino acid CGRP peptide. CGRP is packaged and co-released with substance P in a calcium dependent manner due to depolarization or via activation of TRPA1 or TRPV1 channels.

Calcitonin gene-related peptide is a 37 amino acid peptide, produced by alternative splicing of the calcitonin gene (CALCA) (Amara et al., 1982; Rosenfeld et al., 1983; **Figure 1**). Human CGRP exists in α and β forms, which share 94% structural similarity (Zaidi et al., 1990), with different residues at positions 3, 22, and 25. β-CGRP is transcribed from a separate CALCB gene, which has been proposed to be the result of a duplication of the alpha gene (Alevizaki et al., 1986). Both α-CGRP and β-CGRP have comparable biological roles, but α-CGRP is the principal form and is found in the central and peripheral nervous systems and is the primary subject of this review (Russell et al., 2014), while β-CGRP plays a larger role in enteric transmission (Mulderry et al., 1985). The expression of calcitonin and CGRP mRNA is tissue-specific, and CGRP mRNA is produced by splicing the first three exons to the fifth and sixth exons of CALCA (Amara et al., 1982). The translated CGRP protein then undergoes post-translational modification and protease cleavage to generate the mature peptide. This process is illustrated in **Figure 1**.

### Storage, Release and Metabolism

Calcitonin gene-related peptide is localized throughout the peripheral and central sensory nervous system as well as other locations in the brain (Rosenfeld et al., 1983; Terenghi et al., 1985). Although CGRP is mostly associated with Aδ and C fiber sensory nerves (Gibbins et al., 1985; Lundberg et al., 1985), immunostaining approaches demonstrated the presence of CGRP in association with smooth muscles in the heart and vasculature (Rosenfeld et al., 1983; Franco-Cereceda et al., 1987; Csillik et al., 1993), suggesting the potential sensory and cardiovascular roles of CGRP. GGRP synthesis mainly occurs in the dorsal root ganglion (DRG) (Deng and Li, 2005) where the pro-peptide is then cleaved to the active form and stored in large dense-core vesicles at the sensory nerve terminals, where CGRP is commonly stored and co-released with substance P (Gulbenkian et al., 1986; Brain and Grant, 2004; Schlereth et al., 2016). Exocytosis leading to release is mediated by calcium-dependent pathways following nerve depolarization (Matteoli et al., 1988; Russell et al., 2014). Although release can be mediated in response to pressor substances, such as the sympathetic noradrenergic transmitter and the hypertensive mediator angiotensin (see section: Hypertension), their release is most commonly associated with the activation of these sensory fibers, either by electrical stimulation or by the activation of transient receptor potential (TRP) channels, as shown in **Figure 1** (Escott and Brain, 1993; Russell et al., 2014). TRP channels are a superfamily of non-selective cation-permeable channels, that are each activated by a range of agonists and stimuli (Zheng, 2013). The precise circumstances under which CGRP is released remain under study. Of particular significance to sensory nerves are TRPV1 (transient receptor potential vanilloid 1) and TRPA1 (transient receptor potential ankyrin 1) channels, which are highly co-expressed in CGRP-positive fibers (Kobayashi et al., 2005). TRPV1 is activated by the chili pepper extract capsaicin as well as noxious stimuli such as heat or acidity, acting as transducers of thermal pain (Caterina et al., 1997; Venkatachalam and Montell, 2007), while TRPA1 channels are activated by a distinct subset of agents, including pungent extracts, such as mustard oil and cinnamaldehyde, noxious cold, as well as by endogenous reactive oxygen species and cellular stress mediators (Bandell et al., 2004; Viana, 2016). Both TRPV1 and TRPA1 channels have been shown to trigger CGRP release by increasing intracellular calcium levels *in vitro* (Quallo et al., 2015; Shang et al., 2016; Eberhardt et al., 2017) and *in vivo*, producing neurogenic vasodilation (Pozsgai et al., 2012; Aubdool et al., 2016). In human skin, capsaicin produces a flare that is mediated by CGRP (Van der Schueren et al., 2008). TRPV1 was shown to contribute to cardiovascular pathophysiology under certain conditions, such as high fat diet, but not in a CGRP-dependent manner (Marshall et al., 2013). Moreover, TRPA1 has no observed contribution to blood pressure regulation (Bodkin et al., 2014). The depletion of CGRP positive nerves is possible by repeated TRPV1 agonist application, such as capsaicin and resiniferatoxin, resulting in the desensitization of sensory nerves, which is often used as a blocking strategy (Jancsó et al., 1967; Bánvölgyi et al., 2005). Following its release and action, CGRP has a short half-life, with one study calculating it to have a biphasic clearance with a 6.9-min initial half-life and a 26.4 min slower decay (Kraenzlin et al., 1985). It is metabolized via proteases and possibly other mechanisms (Russell et al., 2014).

### CGRP Source and Plasma Levels

The main source of plasma CGRP is thought to be due to a “spillover” from its release from the perivascular nerve endings, which may contribute to its vasodilatory role (Brain and Grant, 2004). Circulating plasma levels in healthy volunteers are low (in the pg/ml range), which in part is due to the rapid metabolic clearance of plasma CGRP (Kraenzlin et al., 1985). However, CGRP is naturally elevated in pregnancy where it is proposed to regulate utero/placental blood flow and other vascular changes (Dong et al., 2002). Plasma levels are elevated in certain pathological states, such as sepsis (Joyce et al., 1990a), where hypotension poses a major comorbidity. In migraine, only cerebral vessel and ipsilateral jugular vein levels, but not plasma levels, of CGRP are elevated (Goadsby et al., 1990; Edvinsson and Goadsby, 1995; Ashina et al., 2000; Cernuda-Morollón et al., 2015; Messlinger, 2018). Changes in plasma CGRP levels under pathological conditions are further discussed in respective sections. A range of non-neuronal cells have also been shown to synthesize CGRP, including lymphocytes and monocytes (Bracci-Laudiero et al., 2002; Wang et al., 2002; Linscheid et al., 2004). Endothelial cells have also been shown to synthesize and store CGRP in Weibel-Palade bodies where it may play a role in autoregulation of hemodynamics (Ozaka et al., 1997; Doi et al., 2001). Thus, there appear to be multiple sources of CGRP, but the neuronal sources are considered the most important in terms of cardiovascular physiology and pathology and indeed the relevance of some of the cellular sources is unknown.

### The CGRP Receptor

**FIGURE 2:**
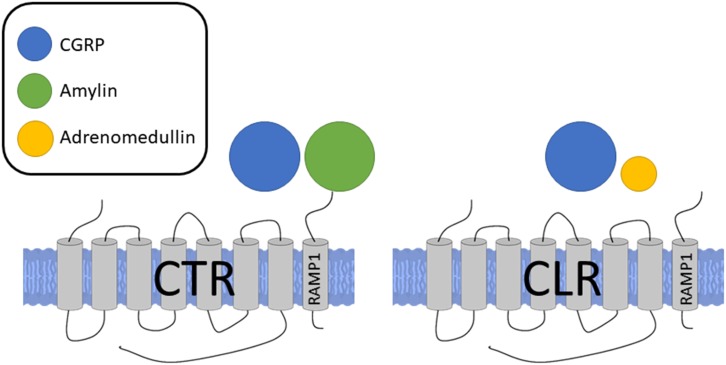
Receptors for CGRP, formed by association of either CTR or CLR with RAMP1. Sizes of different agonists indicate relative potencies at each receptor complex. CGRP and amylin are equally potent at the CTR/RAMP1 receptor complex while CGRP is more potent at the CLR/RAMP1 complex, the classical CGRP receptor.

The biological effects of CGRP are mainly mediated by its associated receptor. The complex nature of the CGRP receptor has been discussed in-depth recently in Hay et al. (2018). Following an intense period of research, it is now realized that the canonical CGRP receptor is a complex of calcitonin receptor-like receptor (CLR), a class B G-protein coupled receptor (GPCR) and a receptor activity modifying protein 1 (RAMP1), a member of the single transmembrane RAMP family (RAMPs 1, 2, or 3), and the receptor component protein (RCP) (McLatchie et al., 1998; Evans et al., 2000; Dickerson, 2013; Russell et al., 2014). RAMP1 is important in the transport of CLR to the plasma membrane where they form a heterodimer to create the full receptor complex and agonist binding (Kuwasako et al., 2006; Booe et al., 2018), while RCP is important in the intracellular G-protein signaling (Evans et al., 2000). However, due to the promiscuous nature of RAMP, it is now thought that CGRP may also be able to signal via other non-canonical receptors of related peptide members, such as the CLR/RAMP3 combination, which is an adrenomedullin receptor (AM_2_) (Qi et al., 2011; Russell et al., 2014) although at a lower potency. Adrenomedullin additionally has some affinity for CGRP receptors, which could account for some of the heterogeneous results and difficulty in early attempts at classifying the CGRP receptor (Moreno et al., 2002). This model has been validated experimentally where CGRP binding correlates well to RAMP1 mRNA levels (Chakravarty et al., 2000). The RAMP proteins regulate the transport of CLR from the endoplasmic reticulum to the plasma membrane (McLatchie et al., 1998) and it has been shown that the cytoplasmic tails of RAMP proteins are crucial for intracellular trafficking of CLR to the membrane. To add to the complexity, more recently it has also been found that association of the calcitonin receptor (CTR) and RAMP1 also forms a CGRP responsive receptor. Although the CTR/RAMP complex was previously described as amylin receptors, it is now shown that CGRP and amylin are equally potent at the CTR/RAMP1 (AMY_1_) receptor (Hay et al., 2018; **Figure 2**). Indeed, the expression of CTR/RAMP1 is suggested to be of potential importance in the sensory trigeminal system, where it may play a role in migraine pain as shown by its expression and histology in rats and humans (Hay et al., 2018). CTRs can promote RAMP translocation to the cell surface and there may be competition between RAMPs, leading to biased formation of one receptor complex over another (Bühlmann et al., 1999). Downstream of the receptor activation, the CGRP receptor complex is coupled to G_αs_ and G_αq_, and the effects of agonist binding are mediated by downstream signaling through these G proteins depending on their sites of action. Using a HEK293 cell line, it was shown that CGRP exhibits biased agonism, favoring the G_αs_ pathway and thus vasodilation (Weston et al., 2016) where CGRP triggers nitric oxide (NO) production in the endothelial cells, acting to relax the underlying vascular smooth muscles, while PKA activation in the vascular smooth muscles also independently mediate NO-independent vasodilation by hyperpolarisation of the smooth muscle cell. Thus, while the pharmacological profile of the CLR receptor expressed on the cell surface is dependent on the RAMP subtype that accompanies it, the pharmacology is complex, potentially involving other CGRP receptors.

## Vasodilator Effects of CGRP

Physiologically, intravenous infusion of CGRP in humans induces a decrease in blood pressure through its vasodilatory effects as well as a positive chronotropic effect, as demonstrated in humans (Struthers et al., 1986), in addition to altering regional blood flow, as observed in human subjects (Jäger et al., 1990). The administration of CGRP by intravenous infusion also increases blood flow to skin (in support of its microvascular potency, resulting in an observable facial flushing) and brain, measured by laser doppler imaging (Jäger et al., 1990). CGRP can also have effects on other organ systems; for example, in the kidneys, CGRP induced relaxation and increase in cAMP levels result in increased blood flow and a subsequent rise in glomerular filtration that has a significant effect on renal hemodynamics (Kurtz et al., 1989; Edwards and Trizna, 1990). CGRP also affects coronary blood flow by its direct action on the vasculature, independent of any systemic reflexes due to the changes in blood pressure or heart rate, as measured in humans by angiography (Ludman et al., 1991). Of note, as discussed above, the vaso-relaxing effects of CGRP do appear to be predominantly at the peripheral level in small vessels rather than large arteries (Marshall et al., 1988; Sekiguchi et al., 1994). The highly potent effects of CGRP on multiple cardiovascular functions highlight its potential contribution in various cardiovascular diseases.

CGRP has a range of regulatory effects that have been studied in rodents, as described below. However, it has also been shown to generally mediate its vasodilator activity independently of cyclo-oxygenase products, such as prostacyclin (Brain et al., 1985), by comparison, CGRP does have a nitric oxide-dependent vasodilator mechanism in certain tissues in a manner that appears neither species or tissue-dependent (Russell et al., 2014). There is also a distinct but reported interaction whereby the CGRP receptor reduces the effects of the potent constrictor agent endothelin through an interaction involving G-protein βγ subunit (Meens et al., 2012).

## CGRP in Cardiovascular Disease

### Hypertension

Despite its role as a vasodilator, there is no consensus on the levels of CGRP in hypertension. In human patients with hypertension, plasma CGRP has been found to be higher (Masuda et al., 1992), unchanged (Schifter et al., 1991), and decreased (Edvinsson et al., 1989; Portaluppi et al., 1992) as reviewed by several groups (including Dong et al., 2002; Smillie and Brain, 2011). It has been suggested that these contradictory observations may be due to differences in sampling and radioimmunoassays used as well as due to the heterogeneity in duration, severity, and treatment of the varying hypertensive populations studied (Bell and McDermott, 1996). However, in patients with secondary hypertension, plasma CGRP levels were decreased after adrenalectomy to treat the underlying condition (Masuda et al., 1992). This would indicate that CGRP is a compensatory response to elevated blood pressure and can become depleted or potentially inhibited as disease progresses (Dong et al., 2002; Smillie et al., 2014).

In CGRP knockout (KO) mice, blood pressure was shown to be significantly elevated in some (Kurihara et al., 2003; Mai et al., 2014), but not all strains (Smillie et al., 2014). The role of CGRP has been studied in a range of different rodent models of hypertension. This includes those that involve raising the levels of pressor agent angiotensin. In one study where baseline blood pressure was not affected in CGRP KO mice, angiotensin administration for up to 28 days led to enhanced hypertension, alongside aortic hypertrophy and decreased endothelial nitric oxide synthase expression (Smillie et al., 2014). In the rat angiotensin-induced hypertension model, the co-administration of subdepressor doses of exogenous CGRP for 6 days significantly reduced blood pressure (Fujioka et al., 1991). However, in a 10-day study, angiotensin increased CGRP receptor expression but not endogenous CGRP levels (Li and Wang, 2005). In one CGRP KO mouse strain, plasma renin was higher in KO mice and AT_1_ blockers were found to have greater effects (Li et al., 2004). Thus, there is good evidence that CGRP plays a protective role against the pathophysiology and onset of hypertension (Smillie and Brain, 2011). This was further shown in the spontaneously hypertensive rat model, where treatment with angiotensin-converting enzyme inhibitors increased the density of CGRP innervation and expression, restoring CGRP nerve function and vasodilator responses (Kawasaki et al., 1999). This adds to the concept that CGRP interacts with RAAS to modulate and maintain blood pressure.

To further understand the mechanisms, deoxycorticosterone (DOC) salt and two-kidney, one-clip (2K1C) rat hypertensive models have been utilized. CGRP expression (mRNA) and plasma levels were elevated, and CGRP_8-37_ or capsaicin depletion of CGRP (to deplete and thus lose/block the CGRP component) led to further increases in the already elevated blood pressure in these models (Supowit et al., 1997; Deng et al., 2003). In spontaneously hypertensive rats and phenol induced hypertensive models, CGRP expression (mRNA) and plasma levels were instead decreased, but CGRP_8-37_ or capsaicin pre-treatment had no effect on blood pressure. Hence, it is clear that the loss of CGRP contributes to the development of hypertension (Supowit et al., 1993; Deng et al., 2004).

It is perhaps not surprising that CGRP interacts indirectly with a range of other vasoactive mediators. Early evidence from the rat mesentery indicated that noradrenaline released from sensory nerves could influence release of CGRP and vice versa; indicating the potential of CGRP to be involved in regulating vascular resistance (Kawasaki et al., 1998). Immunohistochemical staining revealed high concentrations of CGRP immunoreactive fibers in the rat mesenteric artery, the intensity of which decreased after pre-treatment with capsaicin, confirming the expression of CGRP in this vascular bed (Kawasaki et al., 1990). Then, subdepressor doses of CGRP were co-administered with norepinephrine, causing a significant reduction in blood pressure in norepinephrine-induced hypertensive rats (Fujioka et al., 1991). Studies blocking CGRP nerve transmission by CGRP_8-37_ or capsaicin depletion showed increased vasoconstriction caused by periarterial nerve stimulation, further showing that exogenous CGRP can attenuate noradrenergic-induced constriction (Takenaga and Kawasaki, 1999). Together, these studies suggest that CGRP has the ability to inhibit sympathetic nervous activity. Indeed, it is considered that perivascular sensory nerves and sympathetic nerves act alongside each other, in rodents at least, to maintain peripheral vascular tone.

Altogether, previous studies have shown that CGRP plays a protective role in the onset and progression of hypertension in rodents. By comparison, the physiological role of CGRP in the normotensive situations in rodents remains unclear, as some knockout mouse strains are mildly hypertensive, indicating a physiological role, as previously mentioned. Usually, these have deletion in the calcitonin gene, in addition to the CGRP, gene (Smillie and Brain, 2011). However, the cardiovascular effects of CGRP blockade have been investigated in rat models (Juhl et al., 2007; Zeller et al., 2008) which found no change in cardiovascular parameters. Hence the evidence mainly from animal models, to date, indicates that CGRP can interact with key pro-hypertensive systems to counterbalance the onset of cardiovascular disease (see **Table 1**)

**Table 1:** The role and protective effects of CGRP in hypertension, ischemia, and heart failure.

Disease	Role of CGRP	Mechanisms of protection	Evidence in animal models	References
Hypertension	CGRP reduces blood pressure in pathologic states but is not involved in physiological regulation of blood pressure	Modulation of RAAS to maintain blood pressure	Plasma renin is higher in CGRP KO mice	Li et al., 2004
			Treatment with ACE inhibitors on spontaneously hypertensive rats restores CGRP function and responses	Kawasaki et al., 1999
		Inhibition of sympathetic activity reduces blood pressure	In a rat model, CGRP antagonist or capsaicin depletion potentiates vasoconstriction induced by periarterial nerve stimulation	Takenaga and Kawasaki, 1999
			CGRP KO mice had increased hypertension and aortic hypertrophy in an angiotensin II model	Smillie et al., 2014
			αCGRP decreased arterial pressure while CGRP_8-37_ increased it in angiotensin II treated rats	Li and Wang, 2005
Heart failure	CGRP is released in a compensatory manner in response to heart failure and acts in a protective manner	Vasodilation decreases afterload to enhance stroke volume	In a congestive heart failure rat model, CGRP induces vasodilation of blood vessels *in vitro*	Bergdahl et al., 1999
			CGRP infusion in dogs increased coronary flow and decreased coronary resistance and blood pressure	Joyce et al., 1990b
		Positive inotropic effects increase stroke volume and ejection fraction	CGRP infusion in dogs increased cardiac contractility and is blocked by ß receptor antagonists. As isolated myocytes show no response to CGRP, it is suggested CGRP mediates positive inotropy through sympathetic activation	Katori et al., 2005
Ischemia	CGRP is released, by the activation of TRPV1 channels, in ischemia where it exerts protective effects against reperfusion injury and mediates preconditioning	The mechanism of the protective effects of CGRP is unclear. CGRP is purported to play a role in ischemic preconditioning and is protective against reperfusion injury	In a rat model of remote hind limb preconditioning, TRPV and CGRP inhibitors abolished protective effects of preconditioning	Singh et al., 2017
			CGRP protects against ischemia-reperfusion injury in a rat liver *ex vivo* model	Song et al., 2009
			CGRP decreases infarct size in a rat mesenteric artery occlusion model. PKC inhibition abolishes the effect of CGRP, and it is suggested myocardial PKCε activation by CGRP mediates protection	Wolfrum et al., 2005

*Mechanisms by which CGRP exerts its effects and evidence in animal studies is also summarized.*

### Ischemia

CGRP is released endogenously in response to ischemia (Mishima et al., 2011) and is suggested to play a role in preconditioning and protection against reperfusion injury. It has been shown in humans that plasma CGRP expression is upregulated following acute myocardial infarction (Roudenok et al., 2001). In rat models, CGRP induced preconditioning has been shown to be able to protect and attenuate reperfusion injury in heart, brain, and hepatic tissue. These responses were abolished by CGRP_8-37_, indicating that CGRP is a key central mediator of preconditioning and acts in a protective role (Li et al., 1996; Song et al., 2009; Liu et al., 2011). The release of CGRP by TRPV1 is also implicated. In a rat model of ischemia/reperfusion, administration of ruthenium red, a non-selective TRP antagonist, abolished the protective effects of remote limb preconditioning, as measured by infarct size, the release of lactate dehydrogenase, and creatine kinase (Singh et al., 2017). The protective effect is also lost with CGRP_8-37_ administration (Wolfrum et al., 2005). This finding is in line with previous studies that have shown plasma CGRP and mRNA levels to be increased after remote limb ischemic preconditioning, suggesting a cardioprotective mechanism involving TRPV1 mediated CGRP release (Gao et al., 2015). CGRP also protects against ischemia/reperfusion injury in the liver, as measured by alanine aminotransferase and glutamate-lactate dehydrogenase in one study (Song et al., 2009). A summary is shown in **Table 1**. However, the mechanism through which CGRP mediates this protective effect remains to be elucidated.

### Heart Failure

In the heart, CGRP-containing nerves are densely distributed around the coronary arteries, ventricular muscle, and the conduction system, thus ideally placed to play a functional role in the maintenance of cardiac homeostasis (Mulderry et al., 1985; Russell et al., 2014). Reported levels of plasma and tissue CGRP in heart failure, as for hypertension, are confusing and in general, poorly described. One study in children with congenital heart disease found that CGRP levels are higher in those with heart failure compared to healthy controls and levels were also positively correlated with the severity of disease (Hsu et al., 2005). However, in adult patients with chronic congestive heart failure, CGRP levels were found to be lower (Taquet et al., 1992). By comparison, increased CGRP levels in blood plasma have been observed in volume overload heart failure in humans, suggesting that it has the potential to act as a compensatory mechanism (Preibisz, 1993). In an *in vitro* model, the administration of the TRPV1 agonist, capsaicin, caused the release of CGRP which had positive inotropic and chronotropic effects on guinea pig atrium that were subject to tachyphylaxis as well as being abolished by capsaicin pre-treatment, to desensitize the nerve (Lundberg et al., 1984). Similar results were observed in a rat model, where CGRP release by another proposed TRPV1 agonist, rutaecarpine, protects against isoprenaline-induced heart failure by relieving cardiac hypertrophy and cardiac apoptosis. These effects were also attenuated with capsaicin pre-treatment, suggesting that CGRP production and release protects against heart failure (Lundberg et al., 1984; Li et al., 2010). This is in keeping with results in the CGRP KO mouse using a transverse aortic constriction (TAC) model (Li et al., 2013). The vasodilator activity of CGRP is considered to decrease cardiac afterload and improve cardiac function (Struthers et al., 1986). In the guinea pig atrium, CGRP was found to increase the L type calcium current by stimulation of adenylyl cyclase leading to positive inotropy, but the same was not found in ventricular myocytes (Nakajima et al., 1991). This finding is also consistent with earlier studies that showed high binding of radiolabelled CGRP ligands in the rat atrium but not the ventricles (Sigrist et al., 1986) and that cAMP levels in the atria significantly increases following administration of CGRP (Ishikawa et al., 1988). The effects of CGRP on intracellular calcium dynamics also appear to be mediated by the PI3K/Akt pathway. Inhibition of PI3K in rat myocytes *in vivo* abolished CGRP induced increases in calcium release and prolonged calcium sequestration (Al-Rubaiee et al., 2013). A summary is shown in **Table 1**.

The concept that exogenous administration of CGRP may be beneficial has been investigated, mainly using the CGRP peptide which, as discussed, has a short half-life. CGRP administration in humans causes an increase in catecholamine levels in the blood, and the inotropic effects of CGRP have been associated with increased plasma norepinephrine and epinephrine (Tortorella et al., 2001). Moreover, the positive inotropy was blocked by adrenergic antagonists but not the hypotensive and chronotropic actions (Gennari and Fischer, 1985). As heart failure often leads to activation of the baroreceptor reflex and sympathetic activity (Wang et al., 2004), the response to CGRP was separated by ganglionic and adrenergic blockade in one canine study. It was found that ganglionic antagonist hexamethonium did not affect CGRP mediated inotropy and vasodilation but the adrenergic antagonist timolol attenuated the heart response to CGRP (Katori et al., 2005). Thus, sympathetic activation is suggested to be, at least in part, an indirect effect of CGRP which enhances cardiac function through systemic vasodilation. In a rat coronary ligation model, CGRP was found to have a dilatory effect, but the response to CGRP in the basilar artery was attenuated. This led to the suggestion that this would be beneficial in heart failure as arterial perfusion pressure would be maintained despite the decreased cardiac output (Bergdahl et al., 1999). Additionally, in a canine model, CGRP administration increased coronary flow and decreased coronary resistance without affecting heart rate, presumably due to its vasodilatory actions on the coronary vasculature (Joyce et al., 1990b). By comparison, in chronic heart failure, there is attenuation of the adrenergic system. In another canine study, the capacity of sympathetic neurons to release noradrenaline was reduced resulting in reduced sympathetic compensation in heart failure (Cardinal et al., 1996). Additionally, desensitization to catecholamines occurs in multiple points of the adrenoceptor cascade, including decreased distribution and amount of adrenoceptors, decreased agonist affinity, and decreased the activity of adenylyl cyclase (Vatner et al., 1996). Thus, in chronic heart failure, the indirect sympathetic effects of CGRP are likely to be dampened. It has been suggested that CGRP acts indirectly through sympathetic signaling as a compensatory mechanism to increased demand, such as during exercise where CGRP levels are found to be increased (Lind et al., 1996). However, in heart failure where adrenergic transmission becomes downregulated, one possibility may be that CGRP may no longer reduce afterload efficiently to improve cardiac output (Katori et al., 2005). Importantly though, CGRP administration has still been shown to be beneficial in the few human studies carried out to date (Gennari et al., 1990; Anand et al., 1991; Ferrari et al., 1991; Shekhar et al., 1991), so more studies are needed.

## Therapeutic Approaches Involving the CGRP Pathway

### Effects of CGRP Antagonists and Antibodies That Benefit Migraine on the Baseline Regulation of Human Blood Pressure

Recently, various pharmacological tools targeting CGRP have been developed for the treatment of migraine. CGRP antagonists such as BIBN 4096 BS (Olcegepant) have been used in migraine to good effect (Olesen et al., 2004) but were not further developed despite showing efficacy (Yao et al., 2013). Importantly, the safety of these compounds has been studied in single dose (Edvinsson, 2005) and longer-term daily dosing (Ho et al., 2014) and no cardiovascular adverse events were detected. However, the studies were relatively small and short-term, lasting no more than 12 weeks, as most of the treatments for migraine are administered acutely for short periods of time and the timeframes of clinical studies reflect this. Recently, longer studies involving antibodies have also progressed without cardiovascular side effects. In one study specifically examining cardiovascular and hemodynamic effects of CGRP antagonism in humans, no clinically significant changes in blood pressure, heart rate, or ECG were detected over 24 weeks (Bigal et al., 2014). There are currently four monoclonal antibodies, against the CGRP peptide or receptor, undergoing phase 2 or 3 clinical trials. At the time of writing, there has not been any cardiovascular adverse event or significant changes in cardiovascular measurements such as electrocardiogram (ECG) attributable to treatment. The findings of the latest trials are detailed (see **Table 2**). To date, most studies have been performed on normal, healthy subjects and do not address how CGRP blockade may affect a compromised cardiovascular system. Considering the findings in the pre-clinical models consistently showing exacerbation of underlying cardiovascular pathologies with CGRP inhibition it is important to address whether CGRP blockade may exacerbate current cardiovascular disease or increase the severity of cardiovascular events that happen and also whether there is a difference between monoclonal antibodies targeted toward the CGRP receptor or peptide (MaassenVanDenBrink et al., 2016). Emerging evidence begins to take this into account, as a recent clinical study investigated the effect of intravenous erenumab administration in patients with stable angina. Consistently, such acute administration did not show a significant difference in exercise-induced angina or ischemia, consistent with the minimal effect of CGRP inhibition in acute conditions (Depre et al., 2018). However, erenumab is currently the only antibody that blocks the CGRP receptor and the results may not be applicable to inhibition of the CGRP ligand. Indeed, further study on whether chronic CGRP depletion adversely affects subjects with cardiovascular disease will be important to establish cardiovascular safety of these drugs, especially as migraine is associated with an increased risk of cardiovascular disease (Sacco and Kurth, 2014).

**Table 2:** Current monoclonal antibodies undergoing clinical trials and latest findings, in terms of cardiovascular adverse events and changes in cardiovascular perimeters.

Name	Target	Trial	Study length	Findings	Reference
Erenumab (AMG-334)	CGRP receptor	STRIVE: Phase 3 DBPC	6 months	No significant cardiovascular adverse events. Safety profile similar to placebo	Goadsby et al., 2017
		ARISE: Phase 3 DBPC	3 months	No clinically significant cardiovascular events, with no change in electrocardiogram (ECG), hematology, or vital signs	Dodick et al., 2018a
		DBPC study	3 months	No significant cardiovascular events and no change in total exercise time in patients with existing cardiovascular disease	Depre et al., 2018
Eptinezumab (ALD403)	CGRP peptide	Phase 2 DBPC	24 weeks	No significant adverse events related to treatment. No difference in vital signs and ECG	Dodick et al., 2014
Fremanezumab (TEV-48125)	CGRP peptide	Phase 2b DBPC	12 weeks	No significant change in vital signs and ECG	Bigal et al., 2015
		Phase 3 DBPC	12 weeks	No significant change in vital signs and ECG. Most adverse events were due to injection and administration	Dodick et al., 2018b
Galcanezumab (LY2951742)	CGRP peptide	EVOLVE-1 Phase 3 DBPC	10 months	No significant change in vital signs or increases in blood pressure. No cardiovascular adverse events reported	Stauffer et al., 2018

### CGRP Pharmacotherapy

The administration of a CGRP agonist will act to enhance CGRP signaling and may prove to be beneficial in cardio-protection. As discussed above, CGRP plays several roles in cardio-protection against hypertension, ischemia, and heart failure. Thus, it follows that administration of exogenous CGRP may have beneficial effects in these conditions. However, one of the major drawbacks of CGRP is that, as a peptide, it cannot be delivered orally. In addition, it has a short half-life and is rapidly cleared from the blood. Exogenous CGRP has been studied in patients with congestive heart failure, given by continuous intravenous infusion over 24 h, and has been demonstrated to be beneficial to hemodynamic variables (Gennari et al., 1990). However, the effects were lost within 30 min of stopping therapy, concurring with the short half-life (Shekhar et al., 1991). Recently, an acylated αCGRP analog has been synthesized and shown to have a significantly longer half-life of 10.2 h using a diabetes model (Nilsson et al., 2016). The same peptide analog was later investigated in murine models of hypertension and heart failure. It was demonstrated that daily dosing with up to 100 nmol/kg protects against hypertension by attenuating cardiac remodeling, oxidative stress, and reduces blood pressure in an Ang-II model of hypertension. Renal function was also preserved, alongside upregulation of RAMP1. In an abdominal aortic constriction model of heart failure, the CGRP analog attenuated cardiac hypertrophy and apoptosis, demonstrating a beneficial effect of chronic CGRP treatment in heart failure over several weeks (Aubdool et al., 2017). In human tissue, this analog also demonstrated similar pharmacological effects to the native peptide, except for a slightly reduced potency but a longer plasma half-life means that it is potentially useful in research as well as therapy (Sheykhzade et al., 2018). Thus, CGRP analogs or agonism may be a viable pharmacological target for the treatment of cardiovascular diseases and warrants further study.

Future studies should be aimed at understanding the mechanisms involved in the protective effects and the relative contribution of the vasodilator compared with other activities of CGRP described above. It is also hoped that the results targeting the protective effects of CGRP act to incentivise studies into the synthesis of non-peptide CGRP agonists, which could potentially lead to an orally available agonist. This will truly allow the therapeutic potential of CGRP agonists to be evaluated clinically. Meanwhile, the possibility that chronic antagonist and antibody therapy leads to cardiovascular side effects will be evaluated in ongoing experimental medicine studies as well as patient observation of those now being given these new agents to treat migraine.

## Conclusion

Calcitonin gene-related peptide is a potent vasodilator peptide released from sensory nerves. While it does not regulate blood pressure or hemodynamics in healthy conditions, it has cardio-protective activities under pathological states. The protective actions of CGRP have been demonstrated in several rodent models of different cardiovascular diseases. CGRP was found to counteract pro-hypertensive systems to protect against hypertension. It remains unclear how important the vasodilator effects are in this. It is also known from studies including those carried out in humans that CGRP decreases afterload and increases inotropy, which is protective in heart failure. Finally, under ischemic stress, CGRP helps preserve cellular energetics, as well as having anti-apoptotic and anti-inflammatory effects. The effects of CGRP on the different pathologies are summarized in **Table 1**. Ultimately, CGRP is released and confers beneficial protective effects under pathological states.

Currently, CGRP therapy is used in the treatment of migraine, where antagonists and antibody inhibitors have shown good efficacy. No adverse cardiovascular effects of concern have been observed in studies to date. However, many of the safety studies on CGRP antagonists have not examined their effects on subjects with compromised cardiovascular systems. CGRP agonists may have therapeutic use in cardiovascular disorders where they are able to mediate protective actions. However, one of the major hurdles of therapeutic use of CGRP is that, as a peptide, administration and metabolism are issues. Thus, further work on CGRP agonists as therapy for cardiovascular disorders could reveal potential new drugs and provide more options in the treatment of cardiovascular disease.

## Author Contributions

ZK and SDB conceptualized and planned the manuscript. ZK wrote the first draft. All authors reviewed, edited, and approved the final version of the manuscript.

## Conflict of Interest Statement

SDB is a consultant for Eli Lilly.The remaining authors declare that the research was conducted in the absence of any commercial or financial relationships that could be construed as a potential conflict of interest.

## References

[B1] Alevizaki M. Shiraishi A. Rassool F. V. Ferrier G. J. MacIntyre I. Legon S. (1986). The calcitonin-like sequence of the beta CGRP gene. *FEBS Lett.* 206 47–52. 10.1016/0014-5793(86)81338-2 3489641

[B2] Al-Rubaiee M. Gangula P. R. Millis R. M. Walker R. K. Umoh N. A. Cousins V. M. (2013). Inotropic and lusitropic effects of calcitonin gene-related peptide in the heart. *Am. J. Physiol. Heart Circ. Physiol.* 304 H1525–H1537. 10.1152/ajpheart.00874.2012 23585136 PMC3680720

[B3] Amara S. G. Jonas V. Rosenfeld M. G. Ong E. S. Evans R. M. (1982). Alternative RNA processing in calcitonin gene expression generates mRNAs encoding different polypeptide products. *Nature* 298 240–244. 10.1038/298240a0 6283379

[B4] Anand I. S. Gurden J. Wander G. S. O’Gara P. Harding S. E. Ferrari R. (1991). Cardiovascular and hormonal effects of calcitonin gene-related peptide in congestive heart failure. *J. Am. Coll. Cardiol.* 17 208–217. 10.1016/0735-1097(91)90729-S 1987228

[B5] Ashina M. Bendtsen L. Jensen R. Schifter S. Olesen J. (2000). Evidence for increased plasma levels of calcitonin gene-related peptide in migraine outside of attacks. *Pain* 86 133–138. 10.1016/S0304-3959(00)00232-3 10779670

[B6] Aubdool A. A. Kodji X. Abdul-Kader N. Heads R. Fernandes E. S. Bevan S. (2016). TRPA1 activation leads to neurogenic vasodilatation: involvement of reactive oxygen nitrogen species in addition to CGRP and NO. *Br. J. Pharmacol.* 173 2419–2433. 10.1111/bph.13519 27189253 PMC4945766

[B7] Aubdool A. A. Thakore P. Argunhan F. Smillie S.-J. Schnelle M. Srivastava S. (2017). A novel α-calcitonin gene-related peptide analogue protects against end-organ damage in experimental hypertension, cardiac hypertrophy, and heart failure. *Circulation* 136 367–383. 10.1161/CIRCULATIONAHA.117.028388 28446517 PMC5519346

[B8] Bandell M. Story G. M. Hwang S. W. Viswanath V. Eid S. R. Petrus M. J. (2004). Noxious cold ion channel TRPA1 is activated by pungent compounds and bradykinin. *Neuron* 41 849–857. 10.1016/S0896-6273(04)00150-3 15046718

[B9] Bánvölgyi Á Pálinkás L. Berki T. Clark N. Grant A. D. Helyes Z. (2005). Evidence for a novel protective role of the vanilloid TRPV1 receptor in a cutaneous contact allergic dermatitis model. *J. Neuroimmunol.* 169 86–96. 10.1016/j.jneuroim.2005.08.012 16188326

[B10] Bell D. McDermott B. J. (1996). Calcitonin gene-related peptide in the cardiovascular system: characterization of receptor populations and their (patho)physiological significance. *Pharmacol. Rev.* 48 253–288. 8804106

[B11] Bergdahl A. Valdemarsson S. Nilsson T. Sun X. Y. Hedner T. Edvinsson L. (1999). Dilatory responses to acetylcholine, calcitonin gene-related peptide and substance P in the congestive heart failure rat. *Acta Physiol. Scand.* 165 15–23. 10.1046/j.1365-201x.1999.00456.x 10072092

[B12] Bigal M. E. Dodick D. W. Rapoport A. M. Silberstein S. D. Ma Y. Yang R. (2015). Safety, tolerability, and efficacy of TEV-48125 for preventive treatment of high-frequency episodic migraine: a multicentre, randomised, double-blind, placebo-controlled, phase 2b study. *Lancet Neurol.* 14 1081–1090. 10.1016/S1474-4422(15)00249-5 26432182

[B13] Bigal M. E. Walter S. Bronson M. Alibhoy A. Escandon R. (2014). Cardiovascular and hemodynamic parameters in women following prolonged CGRP inhibition using LBR-101, a monoclonal antibody against CGRP. *Cephalalgia* 34 968–976. 10.1177/0333102414527646 24662322

[B14] Bodkin J. V. Thakore P. Aubdool A. A. Liang L. Fernandes E. S. Nandi M. (2014). Investigating the potential role of TRPA1 in locomotion and cardiovascular control during hypertension. *Pharmacol. Res. Perspect.* 2:e00052 10.1002/prp2.52 PMC4186440 25505598

[B15] Booe J. M. Warner M. L. Roehrkasse A. M. Hay D. L. Pioszak A. A. (2018). Probing the mechanism of receptor activity–modifying protein modulation of GPCR ligand selectivity through rational design of potent adrenomedullin and calcitonin gene-related peptide antagonists. *Mol. Pharmacol.* 93 355–367. 10.1124/mol.117.110916 29363552 PMC5832325

[B16] Bracci-Laudiero L. Aloe L. Buanne P. Finn A. Stenfors C. Vigneti E. (2002). NGF modulates CGRP synthesis in human B-lymphocytes: a possible anti-inflammatory action of NGF? *J. Neuroimmunol.* 123 58–65. 10.1016/S0165-5728(01)00475-1 11880150

[B17] Brain S. D. Grant A. D. (2004). Vascular actions of calcitonin gene-related peptide and adrenomedullin. *Physiol. Rev.* 84 903–934. 10.1152/physrev.00037.2003 15269340

[B18] Brain S. D. Williams T. J. Tippins J. R. Morris H. R. MacIntyre I. (1985). Calcitonin gene-related peptide is a potent vasodilator. *Nature* 313 54–56. 10.1038/313054a0 3917554

[B19] Bühlmann N. Leuthäuser K. Muff R. Fischer J. A. Born W. (1999). A receptor activity modifying protein (RAMP)2-dependent adrenomedullin receptor is a calcitonin gene-related peptide receptor when coexpressed with human RAMP1. *Endocrinology* 140 2883–2890. 10.1210/endo.140.6.6783 10342881

[B20] Cardinal R. Nadeau R. Laurent C. Boudreau G. Armour J. A. (1996). Reduced capacity of cardiac efferent sympathetic neurons to release noradrenaline and modify cardiac function in tachycardia-induced canine heart failure. *Can. J. Physiol. Pharmacol.* 74 1070–1078. 10.1139/y96-112 8960400

[B21] Caterina M. J. Schumacher M. A. Tominaga M. Rosen T. A. Levine J. D. Julius D. (1997). The capsaicin receptor: a heat-activated ion channel in the pain pathway. *Nature* 389 816–824. 10.1038/39807 9349813

[B22] Cernuda-Morollón E. Ramón C. Martínez-Camblor P. Serrano-Pertierra E. Larrosa D. Pascual J. (2015). Onabotulinumtoxin a decreases interictal CGRP plasma levels in patients with chronic migraine. *Pain* 156 820–824. 10.1097/j.pain.0000000000000119 25735000

[B23] Chakravarty P. Suthar T. P. Coppock H. A. Nicholl C. G. Bloom S. R. Legon S. (2000). CGRP and adrenomedullin binding correlates with transcript levels for calcitonin receptor-like receptor (CRLR) and receptor activity modifying proteins (RAMPs) in rat tissues. *Br. J. Pharmacol.* 130 189–195. 10.1038/sj.bjp.0702975 10781016 PMC1572027

[B24] Csillik B. Tajti L. Kovács T. Kukla E. Rakic P. Knyihár-Csillik E. (1993). Distribution of calcitonin gene-related peptide in vertebrate neuromuscular junctions: relationship to the acetylcholine receptor. *J. Histochem. Cytochem.* 41 1547–1555. 10.1177/41.10.8245413 8245413

[B25] Deng P.-Y. Li Y.-J. (2005). Calcitonin gene-related peptide and hypertension. *Peptides* 26 1676–1685. 10.1016/j.peptides.2005.02.002 16112410

[B26] Deng P.-Y. Ye F. Cai W.-J. Deng H.-W. Li Y.-J. (2004). Role of calcitonin gene-related peptide in the phenol-induced neurogenic hypertension in rats. *Regul. Pept.* 119 155–161. 10.1016/j.regpep.2004.01.011 15120475

[B27] Deng P.-Y. Ye F. Zhu H.-Q. Cai W.-J. Deng H.-W. Li Y.-J. (2003). An increase in the synthesis and release of calcitonin gene-related peptide in two-kidney, one-clip hypertensive rats. *Regul. Pept.* 114 175–182. 10.1016/S0167-0115(03)00124-1 12832107

[B28] Depre C. Antalik L. Starling A. Koren M. Eisele O. Lenz R. A. (2018). A randomized, double-blind, placebo-controlled study to evaluate the effect of erenumab on exercise time during a treadmill test in patients with stable angina. headache. *Headache* 58 715–723. 10.1111/head.13316 29878340 PMC6001517

[B29] Dickerson I. M. (2013). Role of CGRP-Receptor Component Protein (RCP) in CLR/RAMP function. *Curr. Protein Pept. Sci.* 14 407–415. 10.2174/13892037113149990057 23745704 PMC5019354

[B30] Dodick D. W. Ashina M. Brandes J. L. Kudrow D. Lanteri-Minet M. Osipova V. (2018a). ARISE: a Phase 3 randomized trial of erenumab for episodic migraine. *Cephalalgia* 38 1026–1037. 10.1177/0333102418759786 29471679

[B31] Dodick D. W. Silberstein S. D. Bigal M. E. Yeung P. P. Goadsby P. J. Blankenbiller T. (2018b). Effect of fremanezumab compared with placebo for prevention of episodic migraine: a randomized clinical trial. *JAMA* 319 1999–2008. 10.1001/jama.2018.4853 29800211 PMC6583237

[B32] Dodick D. W. Goadsby P. J. Silberstein S. D. Lipton R. B. Olesen J. Ashina M. (2014). Safety and efficacy of ALD403, an antibody to calcitonin gene-related peptide, for the prevention of frequent episodic migraine: a randomised, double-blind, placebo-controlled, exploratory phase 2 trial. *Lancet Neurol.* 13 1100–1107. 10.1016/S1474-4422(14)70209-1 25297013

[B33] Doi Y. Kudo H. Nishino T. Kayashima K. Kiyonaga H. Nagata T. (2001). Synthesis of calcitonin gene-related peptide (CGRP) by rat arterial endothelial cells. *Histol. Histopathol.* 16 1073–1079. 10.14670/HH-16.1073 11642727

[B34] Dong Y.-L. Vegiraju S. Gangula P. R. Kondapaka S. B. Wimalawansa S. J. Yallampalli C. (2002). Expression and regulation of calcitonin gene-related Peptide receptor in rat placentas. *Biol. Reprod.* 67 1321–1326. 10.1093/biolreprod/67.4.1321 12297551

[B35] Doods H. Hallermayer G. Wu D. Entzeroth M. Rudolf K. Engel W. (2000). Pharmacological profile of BIBN4096BS, the first selective small molecule CGRP antagonist. *Br. J. Pharmacol.* 129 420–423. 10.1038/sj.bjp.0703110 10711339 PMC1571877

[B36] Eberhardt M. Stueber T. de la Roche J. Herzog C. Leffler A. Reeh P. W. (2017). TRPA1 and TRPV1 are required for lidocaine-evoked calcium influx and neuropeptide release but not cytotoxicity in mouse sensory neurons. *PLoS One* 12:e0188008 10.1371/journal.pone.0188008 PMC5687772 29141003

[B37] Edvinsson L. (2005). Clinical data on the CGRP antagonist BIBN4096BS for treatment of migraine attacks. *CNS Drug Rev.* 11 69–76. 10.1111/j.1527-3458.2005.tb00036.x 15867953 PMC6741734

[B38] Edvinsson L. Ekman R. Thulin T. (1989). Reduced levels of calcitonin gene-related peptide (CGRP) but not substance P during and after treatment of severe hypertension in man. *J. Hum. Hypertens.* 3 267–270. 2477545

[B39] Edvinsson L. Goadsby P. (1995). Neuropeptides in the cerebral circulation: relevance to headache. *Cephalalgia* 15 272–276. 10.1046/j.1468-2982.1995.1504272.x 7585922

[B40] Edvinsson L. Villalón C. M. MaassenVanDenBrink A. (2012). Basic mechanisms of migraine and its acute treatment. *Pharmacol. Ther.* 136 319–333. 10.1007/s13311-017-0592-1 22939884

[B41] Edwards R. M. Trizna W. (1990). Calcitonin gene-related peptide: effects on renal arteriolar tone and tubular cAMP levels. *Am. J. Physiol.* 258 F121–F125. 10.1152/ajprenal.1990.258.1.F121 2154123

[B42] Escott K. J. Brain S. D. (1993). Effect of a calcitonin gene-related peptide antagonist (CGRP8-37) on skin vasodilatation and oedema induced by stimulation of the rat saphenous nerve. *Br. J. Pharmacol.* 110 772–776. 10.1111/j.1476-5381.1993.tb13878.x 8242250 PMC2175901

[B43] Evans B. N. Rosenblatt M. I Mnayer L. O. Oliver K. R. Dickerson I. M. (2000). CGRP-RCP, a novel protein required for signal transduction at calcitonin gene-related peptide and adrenomedullin receptors. *J. Biol. Chem.* 275 31438–31443. 10.1074/jbc.M005604200 10903324

[B44] Ferrari R. Panzali A. F. Poole-Wilson P. A. Anand S. (1991). Plasma CGRP-like immunoreactivity in treated and untreated congestive heart failure. *Lancet* 338:1084 10.1016/0140-6736(91)91944-P 1681388

[B45] Franco-Cereceda A. Henke H. Lundberg J. M. Petermann J. B. Hökfelt T. Fischer J. A. (1987). Calcitonin gene-related peptide (CGRP) in capsaicin-sensitive substance P-immunoreactive sensory neurons in animals and man: distribution and release by capsaicin. *Peptides* 8 399–410. 10.1016/0196-9781(87)90117-3 2438668

[B46] Fujioka S. Sasakawa O. Kishimoto H. Tsumura K. Morii H. (1991). The antihypertensive effect of calcitonin gene-related peptide in rats with norepinephrine- and angiotensin II-induced hypertension. *J. Hypertens.* 9 175–179. 10.1097/00004872-199102000-00013 1849535

[B47] Gao Y. Song J. Chen H. Cao C. Lee C. (2015). TRPV1 activation is involved in the cardioprotection of remote limb ischemic postconditioning in ischemia-reperfusion injury rats. *Biochem. Biophys. Res. Commun.* 463 1034–1039. 10.1016/j.bbrc.2015.06.054 26079883

[B48] Gennari C. Fischer J. A. (1985). Cardiovascular action of calcitonin gene-related peptide in humans. *Calcif. Tissue Int.* 37 581–584. 10.1007/BF02554909 3937576

[B49] Gennari C. Nami R. Agnusdei D. Fischer J. A. (1990). Improved cardiac performance with human calcitonin gene related peptide in patients with congestive heart failure. *Cardiovasc. Res.* 24 239–241. 10.1093/cvr/24.3.239 2346957

[B50] Gibbins I. L. Furness J. B. Costa M. MacIntyre I. Hillyard C. J. Girgis S. (1985). Co-localization of calcitonin gene-related peptide-like immunoreactivity with substance P in cutaneous, vascular and visceral sensory neurons of guinea pigs. *Neurosci. Lett.* 57 125–130. 10.1016/0304-3940(85)90050-3 2412189

[B51] Goadsby P. J. Edvinsson L. Ekman R. (1990). Vasoactive peptide release in the extracerebral circulation of humans during migraine headache. *Ann. Neurol.* 28 183–187. 10.1002/ana.410280213 1699472

[B52] Goadsby P. J. Reuter U. Hallström Y. Broessner G. Bonner J. H. Zhang F. (2017). A controlled trial of erenumab for episodic migraine. *N. Engl. J. Med.* 377 2123–2132. 10.1056/NEJMoa1705848 29171821

[B53] Gulbenkian S. Merighi A. Wharton J. Varndell I. M. Polak J. M. (1986). Ultrastructural evidence for the coexistence of calcitonin gene-related peptide and substance P in secretory vesicles of peripheral nerves in the guinea pig. *J. Neurocytol.* 15 535–542. 10.1007/BF01611735 2427663

[B54] Hay D. L. Garelja M. L. Poyner D. R. Walker C. S. (2018). Update on the pharmacology of calcitonin/CGRP family of peptides: IUPHAR review 25. *Br. J. Pharmacol.* 175 3–17. 10.1111/bph.14075 29059473 PMC5740251

[B55] Ho T. W. Connor K. M. Zhang Y. Pearlman E. Koppenhaver J. Fan X. (2014). Randomized controlled trial of the CGRP receptor antagonist telcagepant for migraine prevention. *Neurology* 83 958–966. 10.1212/WNL.0000000000000771 25107879

[B56] Hsu J.-H. Yeh J.-L. Dai Z.-K. Chen I.-J. Wu J.-R. (2005). Increased circulating calcitonin gene-related peptide in congestive heart failure caused by congenital heart disease. *Int. Heart J.* 46 867–875. 10.1536/ihj.46.867 16272777

[B57] Ishikawa T. Okamura N. Saito A. Masaki T. Goto K. (1988). Positive inotropic effect of calcitonin gene-related peptide mediated by cyclic AMP in guinea pig heart. *Circ. Res.* 63 726–734. 10.1161/01.RES.63.4.726 2844436

[B58] Jäger K. Muench R. Seifert H. Beglinger C. Bollinger A. Fischer J. A. (1990). Calcitonin gene-related peptide (CGRP) causes redistribution of blood flow in humans. *Eur. J. Clin. Pharmacol.* 39 491–494. 10.1007/BF00280942 2076743

[B59] Jancsó N. Jancsó-GáBor A. SzolcsáNyi J. (1967). Direct evidence for neurogenic inflammation and its prevention by denervation and by pretreatment with capsaicin. *Br. J. Pharmacol. Chemother.* 31 138–151. 10.1111/j.1476-5381.1967.tb01984.x 6055248 PMC1557289

[B60] Joyce C. D. Fiscus R. R. Wang X. Dries D. J. Morris R. C. Prinz R. A. (1990a). Calcitonin gene-related peptide levels are elevated in patients with sepsis. *Surgery* 108 1097–1101. 2247835

[B61] Joyce C. D. Prinz R. A. Thomas J. X. Fiscus R. R. Wang X. Djuricin G. (1990b). Calcitonin gene-related peptide increases coronary flow and decreases coronary resistance. *J. Surg. Res.* 49 435–440. 10.1016/0022-4804(90)90192-5 2246888

[B62] Juhl L. Edvinsson L. Olesen J. Jansen-Olesen I. (2007). Effect of two novel CGRP-binding compounds in a closed cranial window rat model. *Eur. J. Pharmacol.* 567 117–124. 10.1016/j.ejphar.2007.04.004 17477918

[B63] Katori T. Hoover D. B. Ardell J. L. Helm R. H. Belardi D. F. Tocchetti C. G. (2005). Calcitonin gene-related peptide in vivo positive inotropy is attributable to regional sympatho-stimulation and is blunted in congestive heart failure. *Circ. Res.* 96 234–243. 10.1161/01.RES.0000152969.42117.ca 15591228

[B64] Kawasaki H. Nuki C. Saito A. Takasaki K. (1990). Role of calcitonin gene-related peptide-containing nerves in the vascular adrenergic neurotransmission. *J. Pharmacol. Exp. Ther.* 252 403–409. 1688944

[B65] Kawasaki H. Okazaki M. Nakatsuma A. Mimaki Y. Araki H. Gomita Y. (1999). Long-term treatment with angiotensin converting enzyme inhibitor restores reduced calcitonin gene-related peptide-containing vasodilator nerve function in mesenteric artery of spontaneously hypertensive rats. *Jpn. J. Pharmacol.* 79 221–229. 10.1254/jjp.79.221 10202858

[B66] Kawasaki H. Takenaga M. Araki H. Futagami K. Gomita Y. (1998). Angiotensin inhibits neurotransmission of calcitonin gene-related peptide-containing vasodilator nerves in mesenteric artery of spontaneously hypertensive rats. *J. Pharmacol. Exp. Ther.* 284 508–515. 9454791

[B67] Kobayashi K. Fukuoka T. Obata K. Yamanaka H. Dai Y. Tokunaga A. (2005). Distinct expression of TRPM8, TRPA1, and TRPV1 mRNAs in rat primary afferent neurons with adelta/c-fibers and colocalization with trk receptors. *J. Comp. Neurol.* 493 596–606. 10.1002/cne.20794 16304633

[B68] Kraenzlin M. E. Ch’ng J. L. Mulderry P. K. Ghatei M. A. Bloom S. R. (1985). Infusion of a novel peptide, calcitonin gene-related peptide (CGRP) in man. Pharmacokinetics and effects on gastric acid secretion and on gastrointestinal hormones. *Regul. Pept.* 10 189–197. 10.1016/0167-0115(85)90013-8 3922013

[B69] Kurihara H. Shindo T. Oh-Hashi Y. Kurihar Y. Kuwaki T. (2003). Targeted disruption of adrenomedullin and alphaCGRP genes reveals their distinct biological roles. *Hypertens. Res.* 26(Suppl.), S105–S108. 10.1291/hypres.26.S105 12630819

[B70] Kurtz A. Schurek H. J. Jelkmann W. Muff R. Lipp H. P. Heckmann U. (1989). Renal mesangium is a target for calcitonin gene-related peptide. *Kidney Int.* 36 222–227. 10.1038/ki.1989.183 2550694

[B71] Kuwasako K. Cao Y.-N. Chu C.-P. Iwatsubo S. Eto T. Kitamura K. (2006). Functions of the cytoplasmic tails of the human receptor activity-modifying protein components of calcitonin gene-related peptide and adrenomedullin receptors. *J. Biol. Chem.* 281 7205–7213. 10.1074/jbc.M511147200 16410241

[B72] Li J. Levick S. P. DiPette D. J. Janicki J. S. Supowit S. C. (2013). Alpha-calcitonin gene-related peptide is protective against pressure overload-induced heart failure. *Regul. Pept.* 185 20–28. 10.1016/j.regpep.2013.06.008 23816470

[B73] Li J. Wang D. H. (2005). Development of angiotensin II-induced hypertension: role of CGRP and its receptor. *J. Hypertens.* 23 113–118. 10.1097/00004872-200501000-00020 15643132

[B74] Li J. Zhao H. Supowit S. C. Dipette D. J. Wang D. H. (2004). Activation of the renin-angiotensin system in alpha-calcitonin gene-related peptide/calcitonin gene knockout mice. *J. Hypertens.* 22 1345–1349. 10.1097/01.hjh.0000125409.50839.f1 15201551

[B75] Li J.-Z. Peng J. Xiao L. Zhang Y.-S. Liao M.-C. Li X.-H. (2010). Reversal of isoprenaline-induced cardiac remodeling by rutaecarpine via stimulation of calcitonin gene-related peptide production. *Can. J. Physiol. Pharmacol.* 88 949–959. 10.1139/y10-067 20962894

[B76] Li Y. J. Xiao Z. S. Peng C. F. Deng H. W. (1996). Calcitonin gene-related peptide-induced preconditioning protects against ischemia-reperfusion injury in isolated rat hearts. *Eur. J. Pharmacol.* 311 163–167. 10.1016/0014-2999(96)00426-8 8891596

[B77] Lind H. Brudin L. Lindholm L. Edvinsson L. (1996). Different levels of sensory neuropeptides (calcitonin gene-related peptide and substance P) during and after exercise in man. *Clin. Physiol.* 16 73–82. 10.1111/j.1475-097X.1996.tb00557.x 8867778

[B78] Linscheid P. Seboek D. Schaer D. J. Zulewski H. Keller U. Müller B. (2004). Expression and secretion of procalcitonin and calcitonin gene-related peptide by adherent monocytes and by macrophage-activated adipocytes. *Crit. Care Med.* 32 1715–1721. 10.1097/01.CCM.0000134404.63292.71 15286549

[B79] Liu Z. Liu Q. Cai H. Xu C. Liu G. Li Z. (2011). Calcitonin gene-related peptide prevents blood-brain barrier injury and brain edema induced by focal cerebral ischemia reperfusion. *Regul. Pept.* 171 19–25. 10.1016/j.regpep.2011.05.014 21718723

[B80] Ludman P. F. Maseri A. Clark P. Davies G. J. (1991). Effects of calcitonin gene-related peptide on normal and atheromatous vessels and on resistance vessels in the coronary circulation in humans. *Circulation* 84 1993–2000. 10.1161/01.CIR.84.5.1993 1934374

[B81] Lundberg J. M. Franco-Cereceda A. Hua X. Hökfelt T. Fischer J. A. (1985). Co-existence of substance P and calcitonin gene-related peptide-like immunoreactivities in sensory nerves in relation to cardiovascular and bronchoconstrictor effects of capsaicin. *Eur. J. Pharmacol.* 108 315–319. 10.1016/0014-2999(85)90456-X 2580718

[B82] Lundberg J. M. Hua Y. Fredholm B. B. (1984). Capsaicin-induced stimulation of the guinea-pig atrium. *Naunyn Schmiedebergs Arch. Pharmacol.* 325 176–182. 10.1007/BF00506198 6201751

[B83] MaassenVanDenBrink A. Meijer J. Villalón C. M. Ferrari M. D. (2016). Wiping out CGRP: potential cardiovascular risks. *Trends Pharmacol. Sci.* 37 779–788. 10.1016/j.tips.2016.06.002 27338837

[B84] Mai T. Wu J. Diedrich A. Garland E. M. Robertson D. (2014). Calcitonin gene related peptide (CGRP) in autonomic cardiovascular regulation and vascular structure. *J. Am. Soc. Hypertens.* 8 286–296. 10.1016/j.jash.2014.03.001 24746612 PMC4072204

[B85] Marshall I. Al-Kazwini S. Holman J. Craig R. (1988). Human alpha-calcitonin gene-related peptide (CGRP) is a potent vasodilator in human mesenteric vasculature. *Br. J. Clin. Pharmacol.* 26 691–695. 10.1111/j.1365-2125.1988.tb05306.x 3266556 PMC1386582

[B86] Marshall N. J. Liang L. Bodkin J. Dessapt-Baradez C. Nandi M. Collot-Teixeira S. (2013). A role for TRPV1 in influencing the onset of cardiovascular disease in obesity. *Hypertension* 61 246–252. 10.1161/HYPERTENSIONAHA.112.201434 23150506

[B87] Masuda A. Shimamoto K. Mori Y. Nakagawa M. Ura N. Iimura O. (1992). Plasma calcitonin gene-related peptide levels in patients with various hypertensive diseases. *J. Hypertens.* 10 1499–1504. 10.1097/00004872-199210120-00010 1338081

[B88] Matteoli M. Haimann C. Torri-Tarelli F. Polak J. M. Ceccarelli B. De Camilli P. (1988). Differential effect of alpha-latrotoxin on exocytosis from small synaptic vesicles and from large dense-core vesicles containing calcitonin gene-related peptide at the frog neuromuscular junction. *Proc. Natl. Acad. Sci. U.S.A.* 85 7366–7370. 10.1073/pnas.85.19.7366 3050995 PMC282187

[B89] McLatchie L. M. Fraser N. J. Main M. J. Wise A. Brown J. Thompson N. (1998). RAMPs regulate the transport and ligand specificity of the calcitonin-receptor-like receptor. *Nature* 393 333–339. 10.1038/30666 9620797

[B90] Meens M. J. Mattheij N. J. van Loenen P. B. Spijkers L. J. Lemkens P. Nelissen J. (2012). G-protein βγ subunits in vasorelaxing and anti-endothelinergic effects of calcitonin gene-related peptide. *Br. J. Pharmacol.* 166 297–308. 10.1111/j.1476-5381.2011.01774.x 22074193 PMC3415655

[B91] Messlinger K. (2018). The big CGRP flood - sources, sinks and signalling sites in the trigeminovascular system. *J. Headache Pain* 19:22 10.1186/s10194-018-0848-0 PMC5847494 29532195

[B92] Mishima T. Ito Y. Hosono K. Tamura Y. Uchida Y. Hirata M. (2011). Calcitonin gene-related peptide facilitates revascularization during hindlimb ischemia in mice. *Am. J. Physiol. Heart Circ. Physiol.* 300 H431–H439. 10.1152/ajpheart.00466.2010 21131474

[B93] Moreno M. J. Terrón J. A. Stanimirovic D. B. Doods H. Hamel E. (2002). Characterization of calcitonin gene-related peptide (CGRP) receptors and their receptor-activity-modifying proteins (RAMPs) in human brain microvascular and astroglial cells in culture. *Neuropharmacology* 42 270–280. 10.1016/S0028-3908(01)00176-9 11804624

[B94] Mulderry P. K. Ghatei M. A. Bishop A. E. Allen Y. S. Polak J. M. Bloom S. R. (1985). Distribution and chromatographic characterisation of CGRP-like immunoreactivity in the brain and gut of the rat. *Regul. Pept.* 12 133–143. 10.1016/0167-0115(85)90194-6 3877953

[B95] Nakajima T. Takikawa R. Sugimoto T. Kurachi Y. (1991). Effects of calcitonin gene-related peptide on membrane currents in mammalian cardiac myocytes. *Pflugers Arch.* 419 644–650. 10.1007/BF00370309 1664940

[B96] Nilsson C. Hansen T. K. Rosenquist C. Hartmann B. Kodra J. T. Lau J. F. (2016). Long acting analogue of the calcitonin gene-related peptide induces positive metabolic effects and secretion of the glucagon-like peptide-1. *Eur. J. Pharmacol.* 773 24–31. 10.1016/j.ejphar.2016.01.003 26808305

[B97] O’Connor T. P. van der Kooy D. (1988). Enrichment of a vasoactive neuropeptide (calcitonin gene related peptide) in the trigeminal sensory projection to the intracranial arteries. *J. Neurosci.* 8 2468–2476. 10.1523/JNEUROSCI.08-07-02468.1988 2470872 PMC6569540

[B98] Olesen J. Diener H.-C. Husstedt I. W. Goadsby P. J. Hall D. Meier U. (2004). Calcitonin gene-related peptide receptor antagonist BIBN 4096 BS for the acute treatment of migraine. *N. Engl. J. Med.* 350 1104–1110. 10.1056/NEJMoa030505 15014183

[B99] Ozaka T. Doi Y. Kayashima K. Fujimoto S. (1997). Weibel-Palade bodies as a storage site of calcitonin gene-related peptide and endothelin-1 in blood vessels of the rat carotid body. *Anat. Rec.* 247 388–394. 10.1002/(SICI)1097-0185(199703)247:3<388::AID-AR10>3.0.CO;2-L 9066916

[B100] Paemeleire K. MaassenVanDenBrink A. (2018). Calcitonin-gene-related peptide pathway mAbs and migraine prevention. *Curr. Opin. Neurol.* 31 274–280. 10.1097/WCO.0000000000000548 29432219

[B101] Portaluppi F. Trasforini G. Margutti A. Vergnani L. Ambrosio M. R. Rossi R. (1992). Circadian rhythm of calcitonin gene-related peptide in uncomplicated essential hypertension. *J. Hypertens.* 10 1227–1234. 10.1097/00004872-199210000-00017 1335005

[B102] Pozsgai G. Hajna Z. Bagoly T. Boros M. Kemény Á Materazzi S. (2012). The role of transient receptor potential ankyrin 1 (TRPA1) receptor activation in hydrogen-sulphide-induced CGRP-release and vasodilation. *Eur. J. Pharmacol.* 689 56–64. 10.1016/j.ejphar.2012.05.053 22721614

[B103] Preibisz J. J. (1993). Calcitonin gene-related peptide and regulation of human cardiovascular homeostasis. *Am. J. Hypertens.* 6 434–450. 10.1093/ajh/6.5.434 8390269

[B104] Qi T. Ly K. Poyner D. R. Christopoulos G. Sexton P. M. Hay D. L. (2011). Structure–function analysis of amino acid 74 of human RAMP1 and RAMP3 and its role in peptide interactions with adrenomedullin and calcitonin gene-related peptide receptors. *Peptides* 32 1060–1067. 10.1016/j.peptides.2011.03.004 21402116

[B105] Quallo T. Gentry C. Bevan S. Broad L. M. Mogg A. J. (2015). Activation of transient receptor potential ankyrin 1 induces CGRP release from spinal cord synaptosomes. *Pharmacol. Res. Perspect.* 3:e00191 10.1002/prp2.191 PMC4777244 27022465

[B106] Raffaelli B. Reuter U. (2018). The biology of monoclonal antibodies: focus on calcitonin gene-related peptide for prophylactic migraine therapy. *Neurotherapeutics* 15 324–335. 10.1007/s13311-018-0622-7 29616494 PMC5935651

[B107] Rosenfeld M. G. Mermod J.-J. Amara S. G. Swanson L. W. Sawchenko P. E. Rivier J. (1983). Production of a novel neuropeptide encoded by the calcitonin gene via tissue-specific RNA processing. *Nature* 304 129–135. 10.1038/304129a0 6346105

[B108] Roudenok V. Gutjar L. Antipova V. Rogov Y. (2001). Expression of vasoactive intestinal polypeptide and calcitonin gene-related peptide in human stellate ganglia after acute myocardial infarction. *Ann. Anat.* 183 341–344. 10.1016/S0940-9602(01)80176-X 11508359

[B109] Russell F. A. King R. Smillie S.-J. Kodji X. Brain S. D. (2014). Calcitonin gene-related peptide: physiology and pathophysiology. *Physiol. Rev.* 94 1099–1142. 10.1152/physrev.00034.2013 25287861 PMC4187032

[B110] Sacco S. Kurth T. (2014). Migraine and the risk for stroke and cardiovascular disease. *Curr. Cardiol. Rep.* 16:524 10.1007/s11886-014-0524-1 25059466

[B111] Schifter S. Krusell L. R. Sehested J. (1991). Normal serum levels of calcitonin gene-related peptide (CGRP) in mild to moderate essential hypertension. *Am. J. Hypertens.* 4 565–569. 10.1093/ajh/4.7.565 1873010

[B112] Schlereth T. Schukraft J. Krämer-Best H. H. Geber C. Ackermann T. Birklein F. (2016). Interaction of calcitonin gene related peptide (CGRP) and substance P (SP) in human skin. *Neuropeptides* 59 57–62. 10.1016/j.npep.2016.06.001 27344069

[B113] Sekiguchi N. Kanatsuka H. Sato K. Wang Y. Akai K. Komaru T. (1994). Effect of calcitonin gene-related peptide on coronary microvessels and its role in acute myocardial ischemia. *Circulation* 89 366–374. 10.1161/01.CIR.89.1.366 8281672

[B114] Shang S. Zhu F. Liu B. Chai Z. Wu Q. Hu M. (2016). Intracellular TRPA1 mediates Ca2+ release from lysosomes in dorsal root ganglion neurons. *J. Cell Biol.* 215 369–381. 10.1083/jcb.201603081 27799370 PMC5100290

[B115] Shekhar Y. C. Anand I. S. Sarma R. Ferrari R. Wahi P. L. Poole-Wilson P. A. (1991). Effects of prolonged infusion of human alpha calcitonin gene-related peptide on hemodynamics, renal blood flow and hormone levels in congestive heart failure. *Am. J. Cardiol.* 67 732–736. 10.1016/0002-9149(91)90531-O 2006623

[B116] Sheykhzade M. Abdolalizadeh B. Koole C. Pickering D. S. Dreisig K. Johansson S. E. (2018). Vascular and molecular pharmacology of the metabolically stable CGRP analogue, SAX. *Eur. J. Pharmacol.* 829 85–92. 10.1016/j.ejphar.2018.04.007 29653090

[B117] Sigrist S. Franco-Cereceda A. Muff R. Henke H. Lundberg J. M. Fischer J. A. (1986). Specific receptor and cardiovascular effects of calcitonin gene-related peptide. *Endocrinology* 119 381–389. 10.1210/endo-119-1-381 3013594

[B118] Singh A. Randhawa P. K. Bali A. Singh N. Jaggi A. S. (2017). Exploring the role of TRPV and CGRP in adenosine preconditioning and remote hind limb preconditioning-induced cardioprotection in rats. *Cardiovasc. Drugs Ther.* 31 133–143. 10.1007/s10557-017-6716-3 28194544

[B119] Smillie S.-J. Brain S. D. (2011). Calcitonin gene-related peptide (CGRP) and its role in hypertension. *Neuropeptides* 45 93–104. 10.1016/j.npep.2010.12.002 21269690

[B120] Smillie S.-J. King R. Kodji X. Outzen E. Pozsgai G. Fernandes E. (2014). An ongoing role of α-calcitonin gene–related peptide as part of a protective network against hypertension, vascular hypertrophy, and oxidative stress. *Hypertension* 63 1056–1062. 10.1161/HYPERTENSIONAHA.113.02517 24516108

[B121] Song S. W. Guo K. J. Shi R. Cheng Y. Liu Y. F. (2009). Pretreatment with calcitonin gene-related peptide attenuates hepatic ischemia/reperfusion injury in rats. *Transplant. Proc.* 41 1493–1498. 10.1016/j.transproceed.2009.03.056 19545664

[B122] Stauffer V. L. Dodick D. W. Zhang Q. Carter J. N. Ailani J. Conley R. R. (2018). Evaluation of galcanezumab for the prevention of episodic migraine: the EVOLVE-1 randomized clinical trial. *JAMA Neurol.* 10.1001/jamaneurol.2018.1212 [Epub ahead of print]. PMC6143119 29813147

[B123] Struthers A. D. Brown M. J. Macdonald D. W. R. Beacham J. L. Stevenson J. C. Morris H. R. (1986). Human calcitonin gene related peptide: a potent endogenous vasodilator in man. *Clin. Sci.* 70 389–393. 10.1042/cs0700389 3486086

[B124] Supowit S. C. Ramana C. V. Westlund K. N. Di Pette D. J. (1993). Calcitonin gene-related peptide gene expression in the spontaneously hypertensive rat. *Hypertension* 21 1010–1014. 10.1161/01.HYP.21.6.1010 8505084

[B125] Supowit S. C. Zhao H. Hallman D. M. DiPette D. J. (1997). Calcitonin gene-related peptide is a depressor of deoxycorticosterone-salt hypertension in the rat. *Hypertension* 29 945–950. 10.1161/01.HYP.29.4.945 9095081

[B126] Takenaga M. Kawasaki H. (1999). Endogenous calcitonin gene-related peptide suppresses vasoconstriction mediated by adrenergic nerves in rat mesenteric resistance blood vessels. *Eur. J. Pharmacol.* 367 239–245. 10.1016/S0014-2999(98)00949-2 10078998

[B127] Taquet H. Komajda M. Grenier O. Belas F. Landault C. Carayon A. (1992). Plasma calcitonin gene-related peptide decreases in chronic congestive heart failure. *Eur. Heart J.* 13 1473–1476. 10.1093/oxfordjournals.eurheartj.a060088 1464337

[B128] Terenghi G. Polak J. M. Ghatei M. A. Mulderry P. K. Butler J. M. Unger W. G. (1985). Distribution and origin of calcitonin gene-related peptide (CGRP) immunoreactivity in the sensory innervation of the mammalian eye. *J. Comp. Neurol.* 233 506–516. 10.1002/cne.902330410 2579983

[B129] Tortorella C. Macchi C. Forneris M. Nussdorfer G. G. (2001). Calcitonin gene-related peptide (CGRP), acting via CGRP type 1 receptors, inhibits potassium-stimulated aldosterone secretion and enhances basal catecholamine secretion from rat adrenal gland. *Int. J. Mol. Med.* 8 261–264. 10.3892/ijmm.8.3.261 11494052

[B130] Van der Schueren B. J. Rogiers A. Vanmolkot F. H. Van Hecken A. Depré M. Kane S. A. (2008). Calcitonin gene-related peptide8-37 antagonizes capsaicin-induced vasodilation in the skin: evaluation of a human in vivo pharmacodynamic model. *J. Pharmacol. Exp. Ther.* 325 248–255. 10.1124/jpet.107.133868 18216286

[B131] Vatner D. E. Sato N. Ishikawa Y. Kiuchi K. Shannon R. P. Vatner S. F. (1996). Beta-adrenoceptor desensitization during the development of canine pacing-induced heart failure. *Clin. Exp. Pharmacol. Physiol.* 23 688–692. 10.1111/j.1440-1681.1996.tb01760.x 8886492

[B132] Venkatachalam K. Montell C. (2007). TRP Channels. *Annu. Rev. Biochem.* 76 387–417. 10.1146/annurev.biochem.75.103004.142819 17579562 PMC4196875

[B133] Viana F. (2016). TRPA1 channels: molecular sentinels of cellular stress and tissue damage. *J. Physiol.* 594 4151–4169. 10.1113/JP270935 27079970 PMC4967735

[B134] Wang H. Xing L. Li W. Hou L. Guo J. Wang X. (2002). Production and secretion of calcitonin gene-related peptide from human lymphocytes. *J. Neuroimmunol.* 130 155–162. 10.1016/S0165-5728(02)00221-7 12225897

[B135] Wang W. Zhu G.-Q. Gao L. Tan W. Qian Z.-M. (2004). Baroreceptor reflex in heart failure. *Sheng Li Xue Bao* 56 269–281. 15224137

[B136] Weston C. Winfield I. Harris M. Hodgson R. Shah A. Dowell S. J. (2016). Receptor activity-modifying protein-directed g protein signaling specificity for the calcitonin gene-related peptide family of receptors. *J. Biol. Chem.* 291 21925–21944. 10.1074/jbc.M116.751362 27566546 PMC5063977

[B137] Wolfrum S. Nienstedt J. Heidbreder M. Schneider K. Dominiak P. Dendorfer A. (2005). Calcitonin gene related peptide mediates cardioprotection by remote preconditioning. *Regul. Pept.* 127 217–224. 10.1016/j.regpep.2004.12.008 15680490

[B138] Yao G. Yu T. Han X. Mao X. Li B. (2013). Therapeutic effects and safety of olcegepant and telcagepant for migraine: a meta-analysis. *Neural Regen. Res.* 8 938–947. 10.3969/j.issn.1673-5374.2013.10.009 25206386 PMC4145922

[B139] Zaidi M. Brain S. D. Tippins J. R. Di Marzo V. Moonga B. S. Chambers T. J. (1990). Structure-activity relationship of human calcitonin-gene-related peptide. *Biochem. J.* 269 775–780. 10.1042/bj2690775 2390067 PMC1131654

[B140] Zeller J. Poulsen K. T. Sutton J. E. Abdiche Y. N. Collier S. Chopra R. (2008). CGRP function-blocking antibodies inhibit neurogenic vasodilatation without affecting heart rate or arterial blood pressure in the rat. *Br. J. Pharmacol.* 155 1093–1103. 10.1038/bjp.2008.334 18776916 PMC2597266

[B141] Zheng J. (2013). Molecular mechanism of TRP channels. *Compr. Physiol.* 3 221–242. 10.1002/cphy.c120001 23720286 PMC3775668

